# Factors Associated with Leaving Hospital against Medical Advice among People Who Use Illicit Drugs in Vancouver, Canada

**DOI:** 10.1371/journal.pone.0141594

**Published:** 2015-10-28

**Authors:** Lianping Ti, M-J Milloy, Jane Buxton, Ryan McNeil, Sabina Dobrer, Kanna Hayashi, Evan Wood, Thomas Kerr

**Affiliations:** 1 British Columbia Centre for Excellence in HIV/AIDS, St. Paul’s Hospital, 1081 Burrard Street, Vancouver, BC, Canada V6Z 1Y6; 2 Department of Medicine, University of British Columbia, 2775 Laurel Street, Vancouver, BC, Canada V5Z 1M9; 3 School of Population and Public Health, University of British Columbia, 2206 East Mall, Vancouver, BC, Canada V6T 1Z3; 4 British Columbia Centre for Disease Control, 655 West 12th Avenue, Vancouver, BC, Canada V5Z 4R4; Central South University, CHINA

## Abstract

**Background:**

Leaving hospital against medical advice (AMA) is common among people who use illicit drugs (PWUD) and is associated with severe health-related harms and costs. However, little is known about the prevalence of and factors associated with leaving AMA among PWUD.

**Methods:**

Data were collected through two Canadian prospective cohort studies involving PWUD between September 2005 and July 2011 and linked to a hospital admission/discharge database. Bivariable and multivariable generalized estimating equations were used to examine factors associated with leaving hospital AMA among PWUD who were hospitalized.

**Results:**

Among 488 participants who experienced at least one hospitalization, 212 (43.4%) left the hospital AMA at least once during the study period. In multivariable analyses, factors positively and significantly associated with leaving hospital AMA included: unstable employment (AOR = 1.92; 95% confidence interval [CI]: 1.22–3.03); recent incarceration (AOR = 1.63; 95%CI: 1.07–2.49); ≥ daily heroin injection (AOR = 1.49; 95%CI: 1.05–2.11); and younger age per year younger (adjusted odds ratio [AOR] = 1.04; 95%CI: 1.02–1.06).

**Conclusions:**

We found a substantial proportion of PWUD in this setting left hospital AMA and that various markers of risk and vulnerability were associated with this phenomenon. Our findings highlight the need to address substance abuse issues early following hospital admission. These findings further suggest a need to develop novel interventions to minimize PWUD leaving hospital prematurely.

## Introduction

Leaving hospital against medical advice (AMA) remains a major healthcare challenge that often leads to an array of negative health consequences and related costs. Previous studies have documented elevated rates of readmission among patients who leave hospital AMA [1–3], suggesting that many of these individuals fail to make a full recovery the first time they are treated. The problem of leaving hospital AMA is also associated with high and preventable costs associated with readmission [2], as patients who leave hospital AMA often return with more serious forms of illness that require lengthier stays in hospital than was initially required [3]. Past studies have also indicated an increased risk of mortality among patients who leave hospital AMA compared to those who had a planned discharge [4,5]. It is noteworthy that there may also be mechanisms indirectly (e.g., health-related patient characteristics) related to the acute illness that are associated with these negative health outcomes and thus, may not solely be a direct result of leaving hospital AMA [6].

A growing body of literature indicates that a large proportion of patients with a history of substance use leave hospital AMA [1]. For example, previous research conducted in Vancouver showed that one-quarter of HIV-positive people who inject drugs (PWID) admitted to an inner-city tertiary care hospital were discharged AMA [7]. Other studies have documented that PWID were two to four times more likely to be discharged AMA compared to their non-PWID counterparts [3,7]. From a population health perspective, this is alarming given the known health risks associated with illicit drug use and leaving hospital AMA [8–10].

Despite the growing body of research dedicated to exploring the complex phenomenon of individuals leaving hospital AMA, at present there are few studies focused specifically on people who use illicit drugs (PWUD) beyond identifying them as being at an increased risk. Additionally, the existing research has relied heavily on retrospective data collected from hospital records. This approach has limitations, including a lack of focus on how more proximal individual factors, as well as broader social, structural and environmental factors, may influence leaving hospital AMA among this population [11]. To address these gaps in the literature, we undertook this study to examine the individual, social, structural and environmental factors that influence leaving hospital AMA among PWUD in Vancouver, Canada. With its universal healthcare system, this setting is unique in that it allows for the investigation of factors associated with these outcomes without the confounding effect of explicit financial barriers to essential medical care. It is hoped that the findings from this study may serve to inform the development of interventions that aim to reduce the rate of PWUD leaving hospital prematurely.

## Methods

### Study sample

The Vancouver Injection Drug Users Study (VIDUS) and the AIDS Care Cohort to evaluate Exposure to Survival Services (ACCESS) are two open prospective cohort studies of PWID and PWUD who have been recruited through self-referral, street outreach and word-of-mouth since May 1996. These cohorts have been described in detail previously [12,13]. Briefly, to be eligible for enrollment, participants had to be at least 18 years of age and reside in the greater Vancouver region. The recruitment and follow-up procedures for the two studies are largely identical, allowing for combined analyses, with the only difference being that HIV-positive individuals who use illicit drugs other than cannabis in the month prior to enrollment are followed in ACCESS whereas HIV-negative individuals who injected drugs in the month prior to enrollment are followed in VIDUS. All eligible participants provided written informed consent. At baseline and semi-annually thereafter, participants complete a harmonized interviewer-administered questionnaire and provide blood samples for HIV and HCV testing, as well as HIV disease monitoring. Participants were given a stipend ($20 CAN) at each study visit for their time and transportation. Through a confidential linkage, the cohort data was linked to St. Paul’s Hospital admission/discharge database, which records all admissions, diagnoses and discharge information for each patient who was hospitalized at St. Paul’s Hospital. It is noteworthy that while we linked our cohort data to only one hospital, St. Paul’s Hospital is located in the heart of downtown Vancouver and is known to service the majority of PWUD in this setting [14,15]. Written consent was obtained for all data linkages via a Personal Health Number, which is a unique lifetime government-provided identifier for residents in the province of British Columbia. The study was approved by the University of British Columbia/Providence Health Care’s Research Ethics Board.

The present study was restricted to participants who experienced at least one admission to in-patient care at St. Paul’s Hospital between September 2005 and July 2011. Participants’ first admission to St. Paul’s Hospital during the study period was considered their baseline and they were followed forward from that point onward. Despite past research focusing on patients with a history of injection drug use and leaving hospital AMA, we purposefully included both PWID and PWUD in our study given that PWUD who do not inject are also vulnerable to an array of health harms that require hospitalization [10,16]. Moreover, as in the case of PWID, we hypothesized that PWUD who do not inject are also at risk of leaving hospital AMA. For the present study, we will henceforth use PWUD to denote both injectors and non-injectors.

### Variable selection

The primary outcome of interest for this analysis was having left hospital AMA during the study period, which was ascertained from the St. Paul’s Hospital database. As defined by St. Paul’s Hospital, participants were designated as leaving hospital AMA if they left the hospital without physician permission and did not return within six hours (e.g., if a participant left the hospital and returned seven hours later they were considered as having left the hospital AMA and their subsequent admission would be considered a new admission to the hospital). Participants were also defined as having left hospital AMA if they did not return to hospital within six hours after obtaining physician permission to leave on a pass (unless the physicians authorized a longer period). This definition is consistent with previous research that examined hospital discharge AMA and readmission rates in this setting [17]. We explored a range of individual, social and structural factors that may potentially be associated with leaving hospital AMA, including: age (per year younger); gender (male vs. female); Aboriginal ancestry (yes vs. no) [3,7]; HIV serostatus (positive vs. negative); in a stable relationship, defined as either legally married/common law or having a regular partner (yes vs. no); homelessness (yes vs. no); ≥ daily heroin injection (yes vs. no); ≥ daily cocaine injection (yes vs. no); ≥ daily crystal methamphetamine injection (yes vs. no); ≥ daily prescription opioid injection, defined as injection of either OxyNeo, OxyContin, Percocet, Tylenol 3, Morphine, Dilaudid, Demerol, Methadone, Fentanyl, Hydrocodone, or Talwin (yes vs. no); ≥ daily crack cocaine non-injection (yes vs. no); binge drug use (yes vs. no); need help injecting (yes vs. no); unstable employment, defined as not having a regular job, temporary job or self-employed (yes vs. no); drug dealing (yes vs. no); sex work (yes vs. no); incarceration (yes vs. no); enrollment in methadone maintenance therapy (MMT) (yes vs. no); difficulty finding drug use paraphernalia, including syringes, needles and crack cocaine pipes (yes vs. no); and ever had negative experiences with healthcare professionals (yes vs. no). All these variables were ascertained from the cohort data and referred to the six months prior to each interview unless otherwise indicated.

### Statistical analyses

Bivariable and multivariable statistics were used to identify factors associated with leaving hospital AMA. Since analyses of factors potentially associated with our outcome of interest included serial measures for each subject, we used generalized estimating equations (GEE) for binary outcomes with logit link for the analysis of correlated data to determine factors associated with leaving hospital AMA. These methods provided standard errors adjusted by multiple observations per person using an exchangeable correlation structure. Therefore, data from every participant follow-up visit were considered in this analysis. As a first step, GEE bivariable analyses were conducted to obtain unadjusted odds ratios and 95% confidence intervals for variables of interest. Then, a multivariable model was fit using an *a priori*-defined statistical protocol based on examination of the quasi-likelihood under the independence model criterion (QIC) for GEE and *p*-values [18]. First, a preliminary model was constructed including all variables significantly associated with the outcome in bivariable analyses at *p* < 0.10. Following this, each variable with the highest *p*-value was removed sequentially, with the final model including the set of variables associated with the lowest QIC. We also quantified the severity of multicollinearity using the variance inflation factor (VIF). All *p*-values are two-sided.

## Results

Between September 2005 and July 2011, a total of 488 PWUD had experienced at least one hospitalization at St. Paul’s Hospital and were included in the study: 211 (43.2%) were female and the median age at baseline was 44 years (interquartile range: 38–50 years). In total, there were 1,176 hospital admissions among these individuals, and during the study period, participants were admitted to the hospital between 1 and 30 times per participant. Among these participants, 212 (43.4%) unique individuals left the hospital AMA at least once during the study period. Table 1 shows the characteristics of the study sample stratified by having ever left hospital AMA.

**Table 1:** Sample characteristics stratified by having ever left hospital against medical advice (n = 488).

Characteristic	Total (%) (*n* = 488)	Ever left hospital against medical advice	
		Yes (*%*) (*n* = 212)	No (%) (*n* = 276)
Age (med, IQR)	44 (38–50)	41 (35–47)	45 (40–51)
Male gender	277 (56.8)	109 (51.4)	168 (60.9)
Aboriginal ancestry	177 (36.3)	85 (40.1)	92 (33.3)
HIV-positive serostatus	284 (58.2)	130 (61.3)	154 (55.8)
In a stable relationship*	134 (27.5)	61 (28.8)	73 (26.4)
Homelessness*	163 (33.4)	83 (39.2)	80 (29.0)
Daily heroin injection*	102 (20.9)	53 (25.0)	49 (17.8)
Daily cocaine injection*	44 (9.0)	21 (9.9)	23 (8.3)
Daily crystal methamphetamine injection	15 (3.1)	8 (3.8)	7 (2.5)
Daily prescription opioid injection*	24 (4.9)	11 (5.2)	13 (4.7)
Daily crack cocaine non-injection*	208 (42.6)	109 (51.4)	99 (35.9)
Binge drug use*	207 (42.4)	85 (40.1)	122 (44.2)
Need help injecting*	87 (17.8)	41 (19.3)	46 (16.7)
Unstable employment*	417 (85.5)	186 (87.7)	231 (83.7)
Drug dealing*	150 (30.7)	77 (36.3)	73 (26.4)
Sex work*	69 (14.1)	34 (16.0)	35 (12.7)
Incarceration*	71 (14.5)	45 (21.2)	26 (9.4)
Enrollment in MMT*	219 (44.9)	83 (39.2)	136 (49.3)
Difficulty finding drug use paraphernalia*	113 (23.2)	50 (23.6)	63 (22.8)
Negative experiences with healthcare professionals	76 (15.6)	36 (17.0)	40 (14.5)

IQR: interquartile range; MMT: methadone maintenance therapy

*Refers to the six-month period prior to interview

As indicated in Table 2, in bivariable analyses, factors significantly and positively associated with leaving hospital AMA included: younger age per year younger (odds ratio [OR] = 1.04; 95% confidence interval [CI]: 1.03–1.06); homelessness (OR = 1.51; 95%CI: 1.10–2.08); ≥ daily heroin injection (OR = 1.76; 95%CI: 1.24–2.48); ≥ daily crack cocaine non-injection (OR = 1.43; 95%CI: 1.09–1.89); drug dealing (OR = 1.53; 95%CI: 1.10–2.12); incarceration (OR = 1.90; 95%CI: 1.26–2.85); and unstable employment (OR = 1.79; 95%CI: 1.14–2.78). Enrollment in MMT (OR = 0.73; 95%CI: 0.55–0.98) was negatively associated with the outcome.

**Table 2:** Bivariable and multivariable GEE analysis of factors associated with leaving hospital against medical advice among hospitalized people who use illicit drugs (n = 488).

Characteristic	Unadjusted		Adjusted	
	Odds Ratio (95% CI)	*p*—value	Odds Ratio (95% CI)	*p*—value
**Age**				
(per year younger)	1.04 (1.03–1.06)	<0.01	1.04 (1.02–1.06)	<0.01
**Gender**				
(male vs. female)	0.76 (0.56–1.04)	0.09		
**Aboriginal ancestry**				
(yes vs. no)	1.27 (0.92–1.75)	0.15		
**HIV serostatus**				
(positive vs. negative)	0.99 (0.72–1.37)	0.95		
In a stable relationship*				
(yes vs. no)	0.93 (0.69–1.26)	0.65		
Homelessness*				
(yes vs. no)	1.51 (1.10–2.08)	0.01		
Daily heroin injection*				
(yes vs. no)	1.76 (1.24–2.48)	<0.01	1.49 (1.05–2.11)	0.03
Daily cocaine injection*				
(yes vs. no)	1.07 (0.65–1.74)	0.80		
Daily crystal methamphetamine injection*				
(yes vs. no)	1.87 (0.85–4.15)	0.12		
Daily prescription opioid injection*				
(yes vs. no)	1.19 (0.63–2.26)	0.60		
Daily crack cocaine non-injection*				
(yes vs. no)	1.43 (1.09–1.89)	0.01		
Binge drug use*				
(yes vs. no)	0.87 (0.66–1.14)	0.31		
Need help injecting*				
(yes vs. no)	1.35 (0.94–1.93)	0.11		
Unstable employment*				
(yes vs. no)	1.79 (1.14–2.78)	0.01	1.92 (1.22–3.03)	<0.01
Drug dealing*				
(yes vs. no)	1.53 (1.10–2.12)	0.01		
Sex work*				
(yes vs. no)	1.46 (0.98–2.18)	0.06		
Incarceration*				
(yes vs. no)	1.90 (1.26–2.85)	<0.01	1.63 (1.07–2.49)	0.02
Enrollment in MMT*				
(yes vs. no)	0.73 (0.55–0.98)	0.04		
Difficulty finding drug use paraphernalia*				
(yes vs. no)	1.12 (0.82–1.54)	0.47		
**Negative experiences with healthcare professionals**				
(yes vs. no)	1.21 (0.84–1.75)	0.30		

GEE: generalized estimating equations; CI: confidence interval; MMT: methadone maintenance therapy

*Refers to the six-month period prior to follow-up

Table 2 also presents multivariable analyses of factors associated with leaving hospital AMA. Variables that remained significantly and positively associated with the outcome included: younger age per year younger (adjusted odds ratio [AOR] = 1.04; 95%CI: 1.02–1.06); daily heroin injection (AOR = 1.49; 95%CI: 1.05–2.11); incarceration (AOR = 1.63; 95%CI: 1.07–2.49); and stable employment (AOR = 1.92; 95%CI: 1.22–3.03). The VIF estimate calculated revealed no significant multicollinearity in our final model.

## Discussion

In the present study we found that a substantial proportion of PWUD in our sample had left the hospital AMA at some point during the study period and that various markers of risk and vulnerability were associated with this phenomenon. It is concerning that hospital discharge AMA occurs frequently among this population given the known health-related harms and costs linked to such events, including hospital readmission and mortality [3,5,19]. It is also likely that many PWUD may have left the hospital to maintain their drug use habits and failed to return to the hospital afterward. To address this, a more permissive approach to hospital policies that allow patients to leave for longer periods of time and return for continued treatment without having to be discharged from the hospital AMA might be appropriate. As well, the development and implementation of novel interventions that aim to minimize leaving hospital prematurely among this population is needed.

Findings from our study revealed that high intensity heroin injection was positively associated with leaving hospital AMA. Given the unavailability of illicit opioids in hospital settings as a result of abstinence-based policies in most hospitals, frequent heroin injectors may face severe withdrawal symptoms and as a result, may discharge themselves from hospital AMA to obtain heroin to maintain their drug dependency [20]. The higher odds of leaving hospital AMA among PWUD who are frequent injectors could also be explained by the complexities around treating opioid-dependent patients for pain [21–23]. In particular, studies have documented the provision of inadequate pain management among these individuals [23,24], which may ultimately contribute to an increased risk of leaving hospital AMA to self-manage their pain [25]. To prevent these potential concerns, hospitals may benefit from consulting liaison teams with specialized skills in managing opioid withdrawal and pain. As well, clinical guidelines to treat chronic non-cancer pain specific to PWUD may be useful for healthcare providers to more appropriately treat pain for these individuals [26].

Previous studies have indicated that PWUD with a history of incarceration are less likely to access and utilize healthcare services [27–29]. Our findings add to the existing literature by revealing a positive relationship between recent incarceration and leaving hospital AMA. This may be due to the negative experiences PWUD contend with in correctional settings, including poor relations between inmates and prison health staff that may create mistrust towards healthcare providers among those previously incarcerated [30]. In addition, recent incarceration may be a marker for more unstable addiction or other comorbidities (e.g., mental illness). Given the limited evidence concerning this dynamic, future research should seek to untangle the precise causal relationships underlying these associations. Nevertheless, a number of interventions that seek to build and improve patient-physician relationships should be considered, including educating physicians on how to effectively identify and appropriately address the needs of PWUD patients. In addition, specialized training in addiction medicine among healthcare providers that may help to remove the stigma and discrimination PWUD sometimes experience in hospitals should be a priority.

Our study found that having unstable employment was a risk factor independently associated with leaving hospital AMA. One explanation for our finding may be that being unstably employed is a marker of greater instability and higher intensity substance abuse [31,32], though further research is needed to tease out these dynamics. Nevertheless, previous studies have demonstrated strong links between unstable employment through street-based income generating activities (e.g., drug dealing, sex work, scavenging), greater exposure to the illicit drug scene and substance abuse [31,33]. Thus, another explanation for our finding may be that, among individuals with unstable employment and who engage in street-based income generation activities, being hospitalized and absent from the drug scene could mean not being able to secure funds for drugs and the loss of a job. As a result, people with unstable employment are more likely to leave hospital AMA to maintain both their job and their drug dependency. However, it is noteworthy that while having stable employment has been shown to be associated with various positive outcomes for PWUD [34], there are a number of barriers to obtaining employment and studies have consistently shown low levels of employment among this population [32,35,36].

There are several limitations to this study. First, we cannot infer causation because of the observational nature of the VIDUS and ACCESS studies. Second, we cannot assess the temporality between the explanatory and outcome variables of interest. Third, our sample was not randomly recruited and therefore may not be representative of all local PWUD. On a related note, there may be a selection bias due to the fact that those who were admitted in hospital may be a sample of high-risk PWUD. Fourth, our survey instrument relied on self-report and thus, may be subject to reporting biases. However, our outcome of interest was ascertained from comprehensive administrative records and therefore is not subject to these biases. Fifth, given that the outcome variable was pre-defined by St. Paul’s Hospital, we could not differentiate between patients who completely left hospital AMA and those who returned after a more liberal timeframe (e.g., returned after six hours). As a result, we may have misclassified patients who returned to the hospital after a delayed period of time and continued their care, who may indeed have different outcomes than those who completely left hospital AMA. Lastly, this research project included only one hospital. This limitation would have resulted in an underestimation of hospital use, as some participants may have accessed other hospitals during the study period. However, as noted above, St. Paul’s Hospital is known to service the majority of PWUD in the study [14,37].

In sum, we found that a substantial proportion of hospitalized PWUD in the study sample left hospital AMA. Younger individuals, those who inject heroin with high frequency, those who were previously incarcerated, and those with unstable employment were more likely to have left hospital AMA. Our findings highlight the need to address substance abuse issues soon following admission to hospital. These findings also suggest a need to develop novel interventions to reduce the rate of PWUD leaving hospital prematurely.
